# Glycosylation in Cancer: Interplay between Multidrug Resistance and Epithelial-to-Mesenchymal Transition?

**DOI:** 10.3389/fonc.2016.00158

**Published:** 2016-06-22

**Authors:** Leonardo Marques da Fonseca, Vanessa Amil da Silva, Leonardo Freire-de-Lima, José Osvaldo Previato, Lucia Mendonça-Previato, Márcia Alves Marques Capella

**Affiliations:** ^1^Laboratório de Glicobiologia, Instituto de Biofísica Carlos Chagas Filho, Universidade Federal do Rio de Janeiro, Rio de Janeiro, Rio de Janeiro, Brazil; ^2^Laboratório de P&D em Práticas Integrativas e Complementares, Faculdade de Farmácia, Universidade Federal do Rio de Janeiro, Rio de Janeiro, Rio de Janeiro, Brazil

**Keywords:** multidrug resistance, epithelial-to-mesenchymal transition, cancer, glycosylation, ABC transporters dependent drug resistance

## Abstract

The expression of unusual glycan structures is a hallmark of cancer progression, and their functional roles in cancer biology have been extensively investigated in epithelial-to-mesenchymal transition (EMT) models. EMT is a physiological process involved in embryonic development and wound healing. It is characterized by loss of epithelial cell polarity and cell adhesion, permitting cell migration, and thus formation of new epithelia. However, this process is unwanted when occurring outside their physiological limit, resulting in fibrosis of organs and progression of cancer and metastasis. Several studies observed that EMT is related to the acquisition of multidrug resistance (MDR) phenotype, a condition in which cancer cells acquire resistance to multiple different drugs, which has virtually nothing in common. However, although some studies suggested interplay between these two apparently distinct phenomena, almost nothing is known about this possible relationship. A common pathway to them is the need for glycosylation, a post-translational modification that can alter biological function. Thus, this review intends to compile the main facts obtained until now in these two areas, as an effort to unravel the relationship between EMT and MDR.

## Introduction

One of the major difficulties faced by physicians in any area of medicine is the inability to prevent disease relapse. Unfortunately, this is a common event in several types of cancer, even in those where chemotherapy appeared initially effective. The acquisition of resistance to diverse chemotherapeutic agents by tumor cells is known as multidrug resistance (MDR) phenotype and is still the major cause of death in patients with small cell lung cancer, breast cancer, ovarian cancer, acute leukemia, and others, despite major advances in cancer chemotherapy. For decades, clinical oncologists and researchers dealt with the phenomenon without recognizing its causes.

In 1970, Biedler and Riehm observed that actinomycin-D-resistant Chinese hamster lung cells and P388 leukemia cells were cross-resistant to vinblastine and daunomycin (1). Soon other groups showed that this phenotype lead to resistance to several other amphiphilic compounds, but the phenomenon remained unexplained, until the group of Ling, in 1976, showed that the MDR phenotype of Chinese hamster ovary cells selected for resistance to colchicine was due to a membrane alteration that reduced the rate of intracellular drug saturation. They demonstrated that those drug-resistant cells retained a cell surface glycoprotein of 170 kDa, which was not observed in wild-type cells (2). Since this glycoprotein was present only in cells displaying altered drug permeability, it was named P-glycoprotein (P-gp), ushering in a new era for cancer and chemotherapy researches.

Epithelial cells are usually organized as single or multiple layers of single filed sheets of uniform cells. The individual cells are highly polarized along their base–apex axis and connect to each other through multiple adhesion molecules, insuring the integrity and function of tissues. On the other hand, mesenchymal cells exhibit higher mobility, a significantly lower degree of organization, and no axis polarization. Epithelial-to-mesenchymal transition (EMT) represents a number of changes an epithelial cell must undergo to gain a mesenchymal phenotype. While this model is of paramount importance in several physiological situations, such as embryogenesis and wound healing, the type 3 EMT is well known for its role in cancer invasiveness and metastasis (3–5).

The most important changes that occur during EMT are the loss of E-cadherin with the concomitant increase in N-cadherin expression and metalloproteinases, such as MMP-2 and MMP-9, and increased translocation of β-catenin to the nucleus (6–8). These changes in protein expression are well studied and very much established as important markers for EMT (9–11).

Although there are lot of evidence supporting the role and importance of glycosylation in the acquisition of drug resistance phenotype (12–15) and during the EMT process (16–20), no work has yet demonstrated how the changes in glycosylation may connect these two multifactorial events. Previous studies revealed that TGF-β1, a known EMT inducer (21), as well as the overexpression of Twist (22), a transcription factor able to activate the EMT program (4), promote the appearance of breast epithelial cells with cancer stem cell (CSC) markers (CD44^high^/CD24^low^). Furthermore, Liang and coworkers demonstrated that the emergence of such phenotype was accompanied by changes in the expression of mono- (GM2) and disialylated (GD1a, GD2, and GD3) gangliosides (22). These groundbreaking studies suggest that such differences in cell surface glycosylation may be further used as a therapeutic strategy to combat the advent of CSC, which, besides having potential for initiating tumor growth (23), is also resistant to a wide diversity of antineoplastic agents (24).

It is important to highlight that epigenetic events, which are somatically inherited through cell division, are potential drivers of acquired drug resistance in cancer (25, 26). The high rate of epigenetic changes in cancer cells generates diversity in gene expression patterns that can quickly evolve through drug selection during treatment, leading to the development of acquired resistance (27, 28).

This review will summarize studies about MDR, with a focus on ATP-binding cassette (ABC) transporters, EMT, glycosylation, and the possible relationship among these mechanisms in cancer. We will also discuss epigenetic factors related to MDR phenotype.

## The Multidrug Resistance Phenotype

Much has happened since the discovery that P-gp and other active membrane transporters were shown to be involved in multidrug resistance. In 1990, a 95-kDa membrane protein responsible for anthracycline resistance in MCF-7/AdrVp(100) cells was described (29). Furthermore, using the same cell line, this protein was cloned by Doyle et al. (30), who termed it as breast cancer resistance protein (BCRP). In 1992, another glycoprotein highly expressed in doxorubicin-resistant H69AR cells was identified, cloned, and named multidrug resistance-associated protein (MRP) (31).

At that moment, it became clear that those proteins shared some sequential and functional homology, and all belonged to the ABC superfamily of proteins (32). The ABC proteins are P-type membrane ATPases, which display two highly conserved amino acid sequences located in their nucleotide-binding domain, the Walker A and Walker B motifs, separated by the “ABC signature” motif LSGGQQ/R/KQR (33). With the discovery of several new transporters allocated in the same superfamily, came the need for a simplified nomenclature and thus, P-gp is now referred as ABCB1, BCRP as ABCG2, and MRP as ABCC1. The inventory of human ABC genes contains 49 isolated and identified elements, and it is now known that there are several more ABC proteins, especially from the ABCC subfamily, which are involved in multidrug resistance (34).

**Figure 1:**
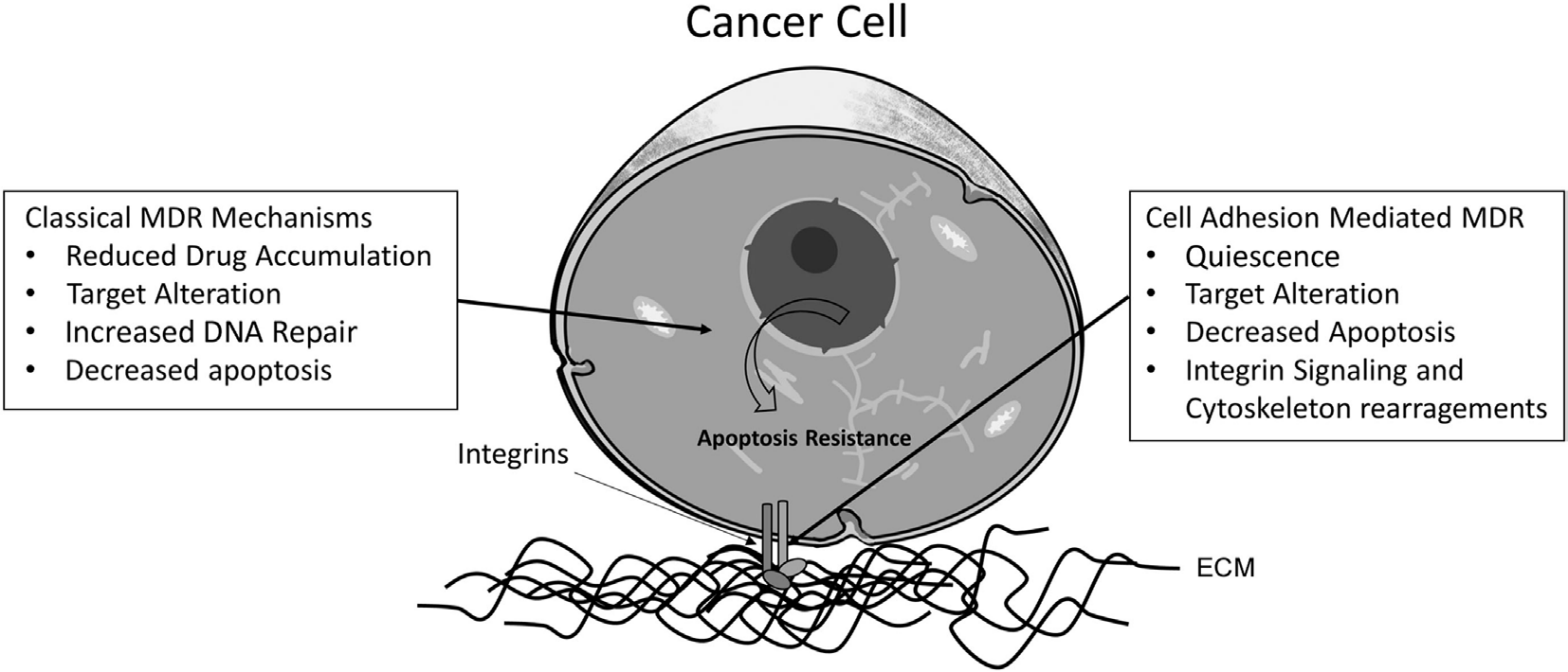
**Proposed classical and cell adhesion-mediated mechanisms for MDR phenotype in cancer cells**. The main mechanisms involved in classical acquired MDR are (1) reduced drug accumulation, mediated by ABC transporters; (2) changes in drug targets, such as variations in the expression or mutations of topoisomerase II; (3) increased activity of enzymes involved in DNA repair mechanisms, such as *O*-6-methylguanine DNA methyltransferase (MGMT); and (4) altered apoptotic signaling, due to mutations in the Bcl-2 family proteins, for instance. Concerning adhesion-dependent mechanisms, also four categories are described: (1) cancer cells can enter a low proliferation state, promoting resistance against drugs affecting the cell cycle, such mechanism could be dependent on E-cadherin adhesion and the cyclin-dependent kinase p27Kip1; (2) cell adhesion mediates changes in the localization of drug targets, such as topoisomerase II, which can be translocated to the cytoplasm *via* an integrin β1-dependent pathway; (3) cell adhesion alters apoptosis signaling pathways. One example is the activation of NF-κB through fibronectin adhesion; and (4) the linker of nucleoskeleton and cytoskeleton (LINC) complex is responsible for binding the nuclear matrix and the cytoskeleton, influencing gene expression, cell morphology, and signal transduction.

Despite the importance of the ABC proteins in MDR, other factors are involved in acquired or constitutive MDR phenotype. A factor that must be considered is the balance between the expression of pro- and anti-apoptotic proteins, responsible for tipping the scale toward cell death or survival. Overexpression of anti-apoptotic proteins, such as Bcl-2, Bcl-xL, and other anti-apoptotic members of the Bcl-2 family (35, 36), reduced expression of pro-apoptotic proteins, such as p-53 (37, 38) and Bax, which are activated by caspase 8 and responsible for permeabilization of the mitochondrial membrane (39, 40), as well as alterations in proteins and extracellular matrix adhesion as integrin-linked kinase (ILK) and αVβ3 integrin (41, 42) are some of the many factors that may contribute to chemotherapy resistance (43). Figure 1 shows some of the main mechanisms involved in MDR phenotype (44–50).

Since the presence of one or more of those ABC proteins in cell membranes is sufficient to trigger the MDR phenotype, they are heavily studied worldwide.

## ABC Proteins Related to MDR: The Glycan Connection

**Figure 2:**
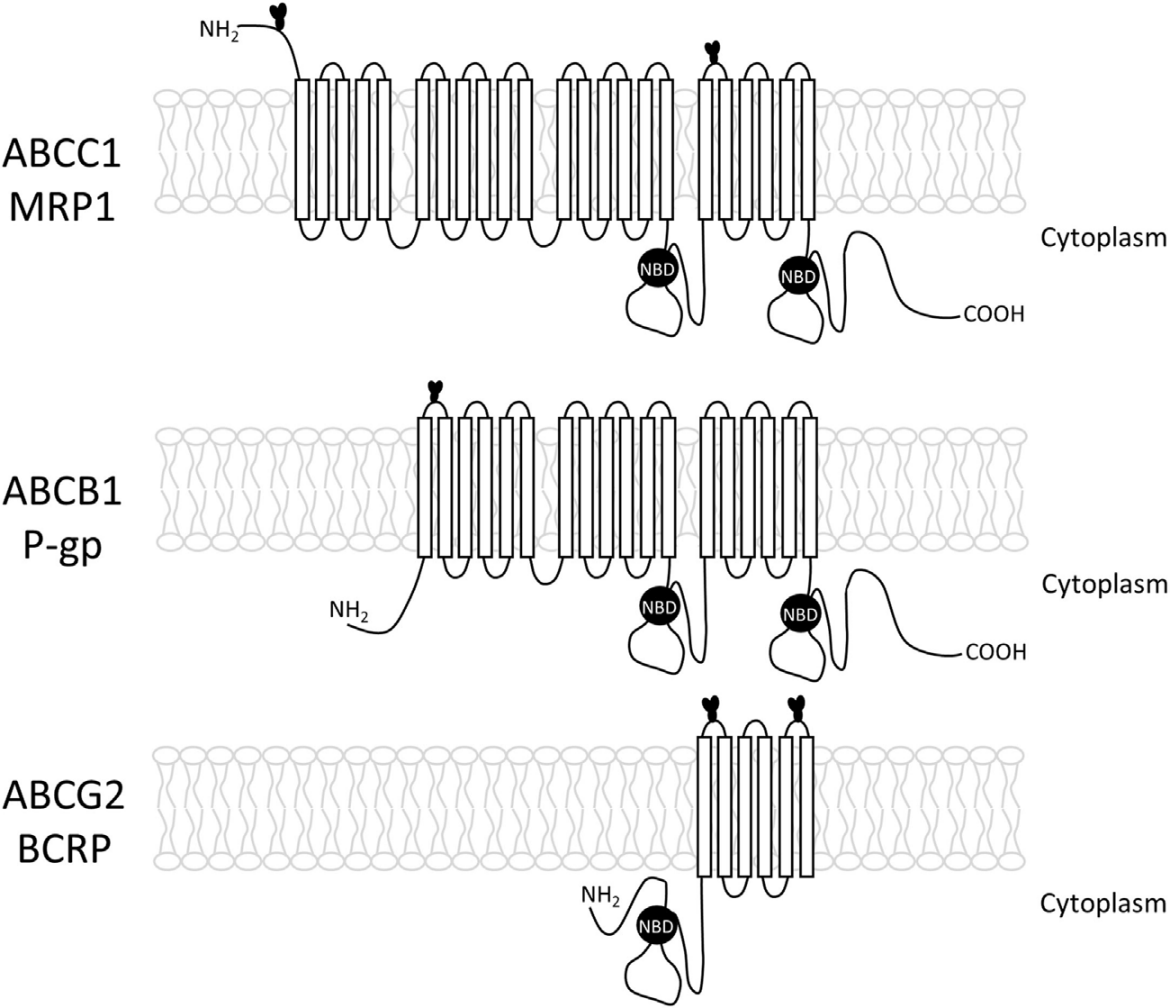
**Structure of the three main ABC transporters (ABCC1, ABCB1, and ABCG2), including transmembrane loops and their glycosylation sites**.

The ABC proteins related to MDR phenotype are glycoproteins; nevertheless, little is known about the importance of glycosylation for their transport activity. During glycosylation, the sugar residues are covalently bound to the protein at asparagine residues for *N*-linked, or at serine, or threonine for *O*-linked glycans (51). The three main ABC proteins related to MDR: ABCB1 (P-gp), ABCC1 (MRP1), and ABCG2 (BCRP) undergo *N*-linked glycosylation (Figure 2). The most studied is ABCB1 or P-glycoprotein, the first protein to be related to the MDR phenotype. This protein is commonly found in cell membranes, but it may be also present at the surface of intracellular organelles, such as the nuclear envelope and lysosomes (52, 53). The human ABCB1 protein contains 10 consensus sequences for *N*-linked glycosylation, but only three of them (N91, N94, and N99), located at the first extracellular loop, are glycosylated. A recent study showed that the topotecan-resistant- and doxorubicin-resistant human ovary carcinoma cell line show ABCB1 expression in both nuclear and plasma membranes, but tunicamycin-treated cells show ABCB1 mainly in the cytoplasm. It seems that tunicamycin, along with other *N*-glycosylation inhibitors, is capable of impairing the activity of ABC transporters by affecting its cellular localization (13). This protein is synthesized as a 140- to 150-kDa protein, and the pathways involved in its glycosylation are still not clear. It has been proposed that the 150-kDa protein is associated with chaperones calnexin and Hsc70 in the endoplasmic reticulum (ER) lumen. Then, ABCB1 moves from *cis* to *trans* Golgi, undergoing final steps of the *N*-glycosylation. After maturation, the protein can be delivered to the membrane in vesicles that move on the cytoskeleton or by an intracellular endosomal system, and transported to the membrane and other cell organelles, including the nucleus (54, 55).

In 1993, Schinkel et al. investigated the significance of the conserved *N*-glycosylation sites present in the putative first extracellular loop of ABCB1. The authors mutated one, two, or all three sites. They observed that the absence of *N*-glycosylation did not alter the level nor the pattern of cross-resistance but dramatically reduced the efficiency with which drug-resistant clones could be generated, suggesting that *N*-glycosylation contributes to proper routing or stability of the protein but not to drug transport itself (54). Two years later, Kramer et al. showed that tunicamycin suppressed ABCB1 activity, observing accumulation of cytostatic drugs into the cells (56).

Draheim et al. (57) used sandwich-cultured rat hepatocytes to characterize the expression and maturation of ABCB1, ABCC2 (MRP2), and ABCG2 proteins, as well as their transport function, over several days. They observed that only fully *N*-glycosylated isoforms of the transporters were associated with functional activity, but they could not determine in what way *N*-glycosylation affected their activity. Apparently, the absence of the glycan trapped the protein in subcellular compartments (57).

Galactose (Gal) and *N*-acetyl glucosamine (GlcNAc) were also found in complex hybrid glycan glycoproteins in a MDR Chinese hamster cell line (58). Greer and Ivey (59), using the uterine sarcoma human cell line MES-SA/D × 5, identified two different complex *N*-glycans in ABCB1 – one high mannose, detected by *Galanthus nivalis* agglutinin (GNA), a lectin that binds specifically to α-1,3 mannose residues and one branched hybrid oligosaccharide – capped with terminal sialic acid units, detected by *Sambucus nigra* (SNA) and *Maackia amurensis* (MAA), lectins with specificity toward α-2,6- and α-2,3-sialic acid residues, respectively. Moreover, *Datura stramonium* (DSA), specific for biantennary oligosaccharides possessing β(1–4)-GlcNAc residues, also recognized the ABCB1 transporter proteins. However, there was no evidence of fucose (Fuc) or mannose (Man), and little or no detected sialic acids attached to ABCB1 glycoproteins in Karnal bunt (KB) cell lines (60). In addition, there is no direct evidence of O-linked glycosylation of ABCB1 (59, 61).

Efforts to demonstrate the glycan structures present in ABCB1 are complicated by the existence of more than 50 known isoforms of this protein, presenting different glycan epitopes. Despite this, it was found that in pediatric brain tumors, the presence of bisecting GlcNAc in human ABCB1 is correlated with tumor progression, showing a potential relevance of glycomes of ABCB1 as tumor markers. Another important finding in this study was the fact that the binding of the PHA-E, a lectin, which has an unusual specificity toward bi- or triantennary galactosylated *N*-glycan with bisecting *N*-acetylglucosamine, was able to subvert chemotherapy resistance in this particular case, through inhibition of P-gp activity (62). Since then, other works have stated the importance of lectins when it comes to drug resistance in cancer. Several studies have pointed out the importance of galectins (a lectin family with an affinity for β-galactosides) in establishing a resistance phenotype (63–65), and many recent papers report the successful use of galectin inhibitors in overcoming resistance (66–68).

## Epithelial-to-Mesenchymal Transition: Differences Between Normal and Cancer Cells

The EMT process can be induced in several cancer cell lines (69–71), usually by TGF-β1 addition or hypoxia, the most common inducers (72).

Among the changes that epithelial cells go through during EMT, alterations in the glycosylation pattern are beginning to attract attention from many groups. One of the most well-known examples is the glycosylation of E-cadherin (73). E-cadherin exhibits four sites for *N*-linked glycan at 554, 566, 618, and 633 asparagine residues, carrying usually a bisected mannosyl core, born from *N*-acetyl glucosaminyl transferase III activity (Gnt-III), which adds a β-1,4 Man to the α-1,6 Man in the *N*-glycan core (74). Such structure stabilizes homophilic interactions between E-cadherin chains and suppresses metastasis (75). Gnt-III competes for its target with Gnt-V, which adds a β-1,6 Man, creating a branched structure that can no longer be modified by Gnt-III. An atypical prevalence of Gnt-V activity over Gnt-III results in tumors with increased mobility and metastatic potential (76).

A more recent paper showed through lectin array and real-time PCR experiments that the expression of the *MAN2A* and *FUCA1* genes, responsible for encoding α-mannosidase 2 and Type 1 α-l-fucosidase, respectively, are reduced during EMT induced by TGF-β in HCV29 bladder cancer cells (77).

A fibronectin isoform, dubbed oncofetal fibronectin (onfFN), for its expression in fetal and cancer tissues, has gained great importance during the last few years. The onfFN differs from other fibronectin isoforms due to the addition of a *O*-glycan epitope (GalNAcα1-*O*-Ser/Thr or Galβ1-3GalNAcα1-*O*-Ser/Thr) to the IICS domain (17). Its expression is significantly increased during TGF-β-induced EMT and is also dependent upon GalNAc-T6/T3 activities, glycosyltransferases that add *N*-acetylgalactosamine (GalNAc) residues to serine or threonine. In fact, knockdown of either GalNAc-T6 or T3 results in EMT inhibition (19). A follow-up study from the same group showed that not only the onfFN is capable, by itself, of inducing EMT in lung cancer cells but it also presents a synergism with TGF-β1, leading to the belief that cells undergoing the transition may secrete onfFN, which once incorporated to the cellular matrix may facilitate the process to other cells exposed to lesser concentrations of TGF-β1 (20). It is important to note that variation in epigenetic patterns lead to heterogeneity, meaning not all cells will undergo EMT at the same time and not all that do undergo will successfully metastasize. Progenitor cell inherent characteristics, intra- and extracellular signaling and epigenetics factors will together determine whether or not a cell will go through EMT and generate metastatic foci (78).

## Epigenetic Modifications and the MDR Phenotype

Among neoplasia-associated epigenetic modifications, alterations in cellular glycosylation have recently received attention as a key factor of neoplastic evolution. Changes in the glycosylation profile seem to not only directly influence cell survival and growth but also assist tumor-induced immunomodulation and subsequent metastasis (79, 80). Similar to that of other genes, the expression of genes encoding glycosyltransferases is regulated through both transcription factors and epigenetic mechanisms (81). As a result, each cell type or tissue has an exclusive set of glycosyltransferases that create specific types of glycan structures (82). As mentioned above, in transformed cells, the expression of glycosyltransferases is often misregulated. In normal mammary epithelial cells, for example, while GnT-V/*MGAT5* is expressed at very low levels, Gnt-III/*MGAT3* is highly expressed (83). However, in breast epithelial cells undergoing EMT, the DNA hypermethylation in promoter CpG islands of *MGAT3* gene inhibits its transcriptional initiation and results in permanent gene silencing (84). Since the methylation status of *MGAT5* did not change along EMT (84), it might be upregulated by the E26 transformation-specific (ETS) transcription factor family through the HER2 pathway, resulting in highly branched *N*-glycan structures on cancer cells (85). It has been demonstrated elsewhere that the unusual glycan structures generated by Gnt-V activity are able to modulate both the EMT process (77, 86, 87) and the gain of an acquired drug-resistant phenotype (12, 88–90).

Dynamic signaling interactions between cancer cells and their stroma in the tumor microenvironment are able to induce a transient and/or resistant state that protect transformed cells by inducing an event known as cell adhesion-mediated drug resistance (CAM-DR) (91). This phenomenon might be triggered by soluble factors such as cytokines, hormones, and growth factors (92, 93) as well as by cell interactions between adjacent cells (94) or extracellular matrix components (ECM) (95, 96). Regarding the protective effects of ECM components, it has been shown that the adhesion of cancer cells to fibronectin, a major ECM component, resulted in increased p27kip1 levels, which correlate with cell cycle arrest and drug resistance (91). Furthermore, adhesion of cancer cells to laminin and collagen IV through beta-1 integrins enhanced tumorigenicity and conferred resistance to apoptosis induced by standard chemotherapeutic agents (97, 98). Since the ECM is composed of various glycoproteins and polysaccharides, and in cancer cell, the enzymatic machinery for glycan biosynthesis is dysregulated, it is believable to speculate that changes in glycosylation of ECM components are capable of modulating both invasiveness and the chemoresistance in cancer cells. New experiments need to be performed to confirm such hypotheses.

## Atypical Glycosylation, EMT, and MDR

Two different studies, using different models reached controversial results suggesting that EMT might not be required for metastasis. Zheng et al. (99), using pancreatic tumors obtained from mice that were knockout for either Snail or Twist showed that in both cases, cancer cells were as likely to cause metastasis as the control tumors, but Snail and Twist deletion made them more sensitive to gemcitabine. In a different work, primary breast tumors from Tri-PY mice (specifically generated to track EMT through fluorescent reporters) overexpressing miR-200, a microRNA capable of regulating *ZEB1* and *ZEB2*, both transcriptional repressors of E-cadherin (100), and although these cells were able to produce lung metastasis when injected into wild-type mice, the metastatic cells have not gone through EMT. Also, in accordance with the work by Zheng et al. (99), the tumors presented increased sensitivity to cyclophosphamide (101). It is important to bear in mind though, that in those studies, the metastatic foci do not originate directly from primary tumors but from cancer cells injected directly into the bloodstream, so the importance of EMT to metastasis end invasion cannot be completely dismissed. A study by Tsou et al. (102) verified that resistance induction on MCF-7 cells with adriamycin leads to both loss of E-cadherin and gain of N-cadherin expressions. Interestingly, such changes are observed alongside other important events, such as loss of Bcl-2 and increased levels of P-gp, which are strongly associated with the acquisition of the MDR phenotype. Such transforming events can be reverted by wortmannin, an inhibitor of PI3K, reducing the resistance to doxorubicin as well as the expression of Snail, Slug, and Twist, all associated with EMT (103, 104). Overexpression of Twist in breast cancer cells has been revealed to promote resistance to radiation in MCF-7 cells through the disruption of p53 function (105), as well as increase invasiveness, motility while mediating resistance to taxol (106–108). Twist overexpression also provokes genome instability in MCF-7 cells (109). Such instability can not only initiate cancer but also affect its progression and prognosis, generating resistance and increasing metastatic potential (110). These results seem to suggest that while EMT may not be entirely necessary to metastasis formation, it does seem to be intimately related to MDR phenotypes. Thus, EMT promoters may prove to be interesting therapeutic targets.

Aside from increased invasiveness, another characteristic conferred to cancer cells by a high expression of Gnt-V is resistance to anoikis through activation of the p21-kinase (PAK1), an upstream mediator of the EGFR pathway (111, 112). Furthermore, overexpression of *MGAT5*, the gene responsible for Gnt-V, in MCF-7 cells, increases resistance to paclitaxel, doxorubicin, and vincristine, while its downregulation achieved a higher sensitivity. Also, in adriamycin-resistant cell line MCF/ADR, expression of the *MGAT5* gene was three times higher than in the parental cell line (89).

In addition to *MGAT5*, Ma et al. (89) also found higher levels of ST6Gal I, a glycosyltransferase responsible for adding sialic acid to Gal residues in a α-2,6 linkage. Using human ovarian cancer cells, Schultz et al. (113) evinced a direct correlation between ST6Gal I expression and resistance to cisplatin. Another study using K562 and K562/ADR human leukemia cell lines showed similar results, while also shedding a light on the mechanism behind the acquired resistance. Silencing the ST6Gal I gene on the resistant cell line lead to the downregulation of the PI3K/AKT pathway and reduced expression of both MRP1 and P-gp, while its overexpression in the parental cells achieved the opposite results (114). The PI3K activation was already correlated to the MDR phenotype, since the pharmacological inhibitor LY294002 is capable of increased doxorubicin accumulation in HT29RDB colon carcinoma cells by reducing MRP1 expression in the membrane and leading to apoptosis (115). A recent study by Meng et al. (116) also correlates ST6Gal I activity to increased mobility and invasiveness of osteosarcoma cells. The authors also discovered that silencing the sialyltransferase decreased expression of N-cadherin and metalloproteinases 2 and 9, as well as other EMT markers. Also, the PI3K pathway plays a role in EMT process in different cancer cell types (117, 118).

Another study by Ma and collaborators (119) using different resistant leukemia cell lines, along with their parent cells, has uncovered important differences regarding sialylation patterns between adriamycin-resistant cells and their non-resistant counterparts. Expression of the ST8Sia IV sialyltransferase was elevated in the HL60-ADR, U937-ADR, and NB4-ADR cell lines, and its knockdown provoked a reduction in the resistance level. On the other hand, overexpression of the *ST8Sia IV* gene in the HL-60 parental cell line leads to increased drug resistance. The changes in resistance level are related to alteration in the expressions of both ABCB1 and ABCC1 drug transporters, through the PI3K/Akt pathway. This type of sialyltransferase is involved with polysialic chains and this particular enzyme is essential to the expression of the neural cell adhesion molecule (NCAM-1). NCAM-1 is indeed involved with chemoresistance mechanisms, but paradoxically, its expression has been confirmed to be decreased in resistant neuroblastoma cell lines exposed to vincristine or doxorubicin when compared to the parental cells (120, 121). The upregulation of NCAM-1 is also linked to the decrease of E-cadherin expression during EMT, being important for the formation of focal adhesion points and integrin-dependent cell mobility (122). Also, its polysialylation through ST8Sia 2 or ST8Sia 4 activity has been shown to be essential to the onset EMT in NMuMG murine cells, a common model for EMT studies (123).

Surprisingly, the same study (119) reveals opposite results for ST3Gal V, with its knockdown being associated with increased resistance to adriamycin, paclitaxel, and vincristine, while its overexpression results in lower resistance levels for the tested drugs. The observed results for the two sialyltransferases (ST8Sia and ST3Gal V) are diametrically opposed, down to the changes in ABCC1 and ABCB1 expression and the involvement of PI3K/Akt signaling pathway, which has been well established as important to drug resistance mechanisms as well as tumor proliferation (115, 124, 125). ST3Gal V is responsible for the synthesis of GM3, a ganglioside and common precursor of almost all gangliosides, especially from the ganglio-series, by adding a α-2,3-sialic acid to lactosilceramide (126). It has been demonstrated before that the glucosylation of ceramide and consequent increase in the content of glycosphingolipids leads to an increase in ABCB1 expression though the activation of Src (127). GM3 itself is thought to influence the level of ABCB1 phosphorylation, which in turn increases drug transport (128). A study by Mathow et al. (129) showed that repression of the ST3Gal V gene results in increased susceptibility disruption of cell adhesion by TGF-β signaling (129), while its upregulation *via* Zeb1 leads to increased cell–cell adhesion.

Fucosylation is another well-studied aspect of glycosylation, when speaking of cancer glycophenotypes. It has been described that fucosyltransferase 4 (Fut4), Fut6, and Fut8 are increased in hepatocellular carcinoma cells exhibiting a MDR phenotype dependant upon the PI3K/Akt pathway. Furthermore, reducing fucosyltransferases expression leads to abrogation of the resistance phenotype (130). Fut4 also appears to play an important role in EMT. A study by Yang et al. (131) confirms that inhibiting the expression of the Fut4 in breast cancer cells leads to a reduced expression of EMT markers, such as N-cadherin and vimentin, as well as an inhibition of the PI3K/Akt pathway. A more recent study sheds more light into the involvement of fucosylation. Using two different breast cancer cell lines, the authors established that miR-224-3p, through the downregulation of the *FUT4* gene, is capable of reverting the MDR phenotype and of increasing sensitivity to taxol, vincristine, and doxorubicin (12). It is also worth considering that in addition to the PI3K/Akt, Fut4 also activates NF-κB signaling, which just like PI3K is also involved in both resistance phenotype by inducing Pg-P expression (132) and EMT signaling (133).

Looking over the data, it is easy to see that not only many of the glycosylation changes represent common points between cells undergoing EMT and cells that have acquired a MDR phenotype but also such changes are often necessary for both EMT and MDR. To establish such connections is not only important to understand the events surrounding metastasis and drug resistance but also to develop ways to circumvent them.

## Conclusion

Despite the abundance of data showing that all ABC proteins are heavily *N*-glycosylated, to this date, there is little evidence on how, or if at all, changes in these glycan epitopes are capable of affecting their transport activity. The exception being for studies using broad glycosylation inhibitors that attest the importance of glycosylation to protein localization within the cell.

There is, however, a plethora of data demonstrating that aberrant glycosylation occurring during cancer development affects among other things, drug resistance. Many groups have successfully identified, despite some contradictions that can be ascribed to the different cell lines and chemotherapy drugs used, a number of glycosylation changes that commonly appear during several resistance inducing protocols, such as higher levels of α-2,6-sialic acid units and altered fucosylation. Many such alterations are also commonly observed in cells undergoing EMT.

Given the increasing number of research groups dedicated to discovering glycosylation patterns common to different resistant cancer cell lines, it will be interesting to see the effect that these discoveries will have on cancer diagnosis, prognosis, and treatment in the next few years, as new biomarkers and therapeutic targets will be discovered. Certainly, these findings might be used to feed further actions to tackle the metastatic and the drug resistance phenotypes, the two main obstacles in the war against cancer. It is also becoming clearer that EMT and chemotherapy resistance not only walk hand in hand but also are joined by glycosylation.

## Author Contributions

LF, VS, and MC contributed to writing the manuscript; LF-d-L, JP, and LM-P reviewed the manuscript; LF, VS, and LF-d-L contributed to the preparation of the figures. All authors approved the final version of the manuscript.

## Conflict of Interest Statement

The authors declare that the research was conducted in the absence of any commercial or financial relationships that could be construed as a potential conflict of interest.
